# MicroRNA-Dependent Targeting of RSU1 and the IPP Adhesion Complex Regulates the PTEN/PI3K/AKT Signaling Pathway in Breast Cancer Cell Lines

**DOI:** 10.3390/ijms21155458

**Published:** 2020-07-30

**Authors:** Yong-Chul Kim, Mary L. Cutler

**Affiliations:** 1 Department of Pathology, Uniformed Services University of the Health Science, Bethesda, MD 20814, USA; yongchul.kim.ctr@usuhs.edu; 2 Murtha Cancer Center, Walter Reed National Military Medical Center, Bethesda, MD 20814, USA

**Keywords:** micro RNA, RSU1, PINCH1, LIMS1, PTEN

## Abstract

(1) Background: The microRNA (miR)-directed control of gene expression is correlated with numerous physiological processes as well as the pathological features of tumors. The focus of this study is on the role of miRs in the regulation of RSU1 and proteins in the IPP (integrin linked kinase, PINCH and parvin) complex. Because the IPP adaptor proteins link β integrins to actin cytoskeleton, and the RSU1 signaling protein connects the complex to the activation of cJun, ATF2 and the transcription of PTEN, their reduction by miRs has the potential to alter both adhesion and survival signaling. (2) Methods: Multiple database analyses were used to identify miRs that target RSU1 and PINCH1. miR transfection validated the effects of miRs on RSU1, PINCH1 and downstream targets in breast cancer cell lines. (3) Results: The miRs targeting RSU1 mRNA include miR-182-5p, -409-3p, -130a-3p, -221-3p, -744-5p and -106b-5p. Data show that miR-182-5p and -409-3p reduce RSU1, PINCH1 and inhibit the ATF2 activation of PTEN expression. miR-221-3p and miR-130a-3p target RSU1 and PINCH1 and, conversely, RSU1 depletion increases miR-221-3p and miR-130a-3p. (4) Conclusions: miRs targeting RSU1 and PINCH1 in mammary epithelial or luminal breast cancer cell lines reduced RSU1 signaling to p38 MAP kinase and ATF2, inhibiting the expression of PTEN. miR-221-3p, known to target PTEN and cell cycle regulators, also targets RSU1 and PINCH1 in luminal breast cancer cell lines.

## 1. Introduction

MicroRNAs, miRs, are small, non-coding RNAs (~22 nt) that negatively regulate protein-coding mRNAs at the post transcriptional level and inhibit target gene expression. miRs have essential roles in many normal cell functions including cell growth, proliferation, differentiation, migration and apoptosis. Increasing evidence supports a role for miRs in cancer development, progression and metastasis [1,2]. Hence, they have potential as diagnostic biomarkers and miR-based anticancer targeted therapies [3].

Among the mRNAs targeted by miRs are those that encode the proteins necessary for cell adhesion and migration. Part of the integrin–actin network is regulated by the IPP complex, which is made up of integrin linked kinase (ILK), PINCH, Parvin and RSU1 [4]. Multiple studies have identified the role for these proteins in the adhesome [5,6]. The complex binds to the cytoplasmic domain of the β1 integrin through the adaptor ILK and connects integrins to the actin cytoskeleton via parvin. PINCH1/LIMS1 is a LIM domain adaptor that binds ILK and RSU1. RSU1 is a 33 kDa leucine rich repeat protein that localizes to focal adhesions though interactions with PINCH1 and functions as a link to downstream signaling. The integrins and Epidermal Growth Factor (EGF) receptor induce adhesion, spreading and migration in non-transformed MCF10A mammary epithelial cells in a process that requires RSU1 [7,8]. RSU1 contributes to integrin-dependent cell adhesion and migration via both PINCH1 stabilization and effects on a signaling pathway that includes MKK4-JNK-p38-ATF2 [8,9,10,11]. RSU1 levels modulate the activation of ATF2 and cJun, which coordinately regulate PTEN expression and PI3K/AKT activity, an important component and regulatory element in cell migration as well as survival [11]. ILK also promotes AKT activation and PINCH1 blocks AKT dephosphorylation by PP1α [12,13,14].

The contribution of miRs to the regulation of RSU1 and the IPP complex in carcinogenesis is not entirely understood. RSU1 is one of the major targets of miR-409-3p/5p. The miR-409-3p/5p-dependent depletion of RSU1 occurs following the epithelial uptake of stromal-produced exosomes in early prostate tumors; the use of miR-409 antagomiRs restored the RSU1 protein and decreased bone metastasis in a mouse model of aggressive prostate cancer [15,16]. RSU1 expression was blocked by miR-182 in lung metastasis of mouse fibrosarcomas, as well as in a breast cancer cell line [17,18], and miR-629 targeted RSU1 in cervical cancer thereby decreasing the sensitivity to a therapeutic agent [19]. miR-625 targets ILK and suppresses invasion and metastasis, and miR-542-3p, which inhibits tumor progression, also targets ILK [20,21]. Collectively, these reports suggest that the miR-dependent regulation of RSU1 and the IPP proteins contribute to tumor progression and metastasis. Therefore, it is important to identify the relevant miRs targeting the components of this complex. Our previous work has identified the role of RSU1 in mammary epithelial and breast cancer cell lines. Hence, the objective of this work is to determine which miRs target RSU1 in mammary epithelial cells and if the miR-targeting of RSU1 also disrupts the signaling pathways linked to RSU1 and the IPP complex.

In our current study, we identify the miRs targeting RSU1 and determine the expression pattern of the miRs across a collection of breast cancer cell lines. The study examines the question of whether the miR-targeting of RSU1 produces the same alterations in signaling previously detected by the siRNA-mediated depletion of RSU1. This is based on the hypothesis that targeting RSU1 by miRs will alter PTEN expression as previously reported for RSU1 depletion by siRNA. The results confirm this and, furthermore, indicate that miR-221-3p and -130a-3p, which target PTEN, are also linked to the regulation of both RSU1 and PINCH1. These data support a novel mechanism for the miR-dependent regulation of PTEN signaling in mammary epithelial cells by targeting RSU1 and the IPP adhesion complex.

## 2. Results

### 2.1. Identification of the miRs Predicted to Target RSU1 and the Validation of Their Function in Human Mammary Epithelial Cells

This study aims to dissect the contribution of miR regulation to RSU1 expression and function. To identify the miRs targeting RSU1 expression, we initially used a sequence-based analysis to identify the miRs predicted to bind RSU1 RNA. Three common tools for the prediction of miR binding were employed. The results from these algorithms were similar and showed considerable overlap (Table 1). These include the algorithms: miRanda (mirSVR scale values < 0.1 predict higher possibility of targeting), MicroT-CDS (0.7–1 or 0–1 scores predict a higher possibility of targeting in the case of the miTG score), and TargetScan (Context+++ score is to be most negative and probability of conserved targeting (Pct) as prediction scores). The same approach was employed to identify PINCH1-targeting miRs (Supplementary Table S2).

**Figure 1:**
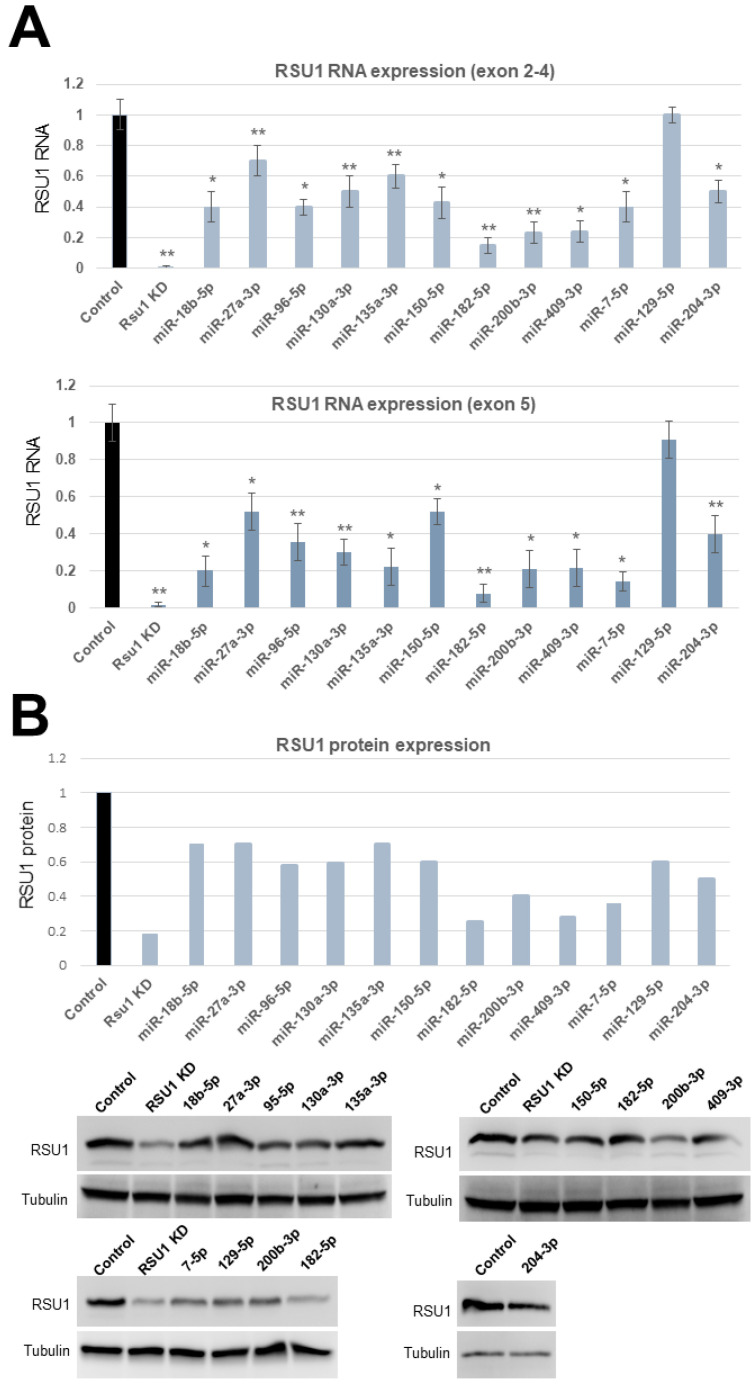
The validation of microRNAs (miRs) targeting RSU1 RNA and protein in MCF10A cells. (**A**). The miRs predicted to target RSU1 were transfected into MCF10A cells to determine the effect on the level of RSU1 RNA. RNA from cells transfected with miR-18b-5p, -27a-3p, -96-5p, -130a-3p, -135a-3p -150-5p, -182-5p, -200b-3p, -409-3p, -7-5p, -204-3p and -129-5p were compared to the RNA from control (non-targeting miR)-transfected cells for RSU1 RNA by quantitative real-time PCR using RSU1 primers for exons 2–4 (top panel) and exon 5 (lower panel). RSU1 KD is the transfection of RSU1-specific siRNA. Values represent the means from triplicate samples normalized to 18S ± S.D. * *p* < 0.05 and ** *p* < 0.01 versus control. Control RNA levels were set to 1. (**B**). An immunoblot analysis was performed with total lysates from the miR-transfected MCF10A cells to detect the levels of RSU1 expression by Western blot analysis. Note that the lysates from siRNA-mediated RSU1 depleted cells serve as the positive control for RSU1-targeting in the MCF10A cells. Blots were quantified as described in Materials and Methods and normalized to α-tubulin protein which served as an internal control. In the cases where the miR was transfected more than once (miR-200b-3p and miR-182-5p), both images are shown and the values for fold change were averaged.

Based on this analysis we focused on miRs -7-5p, -18b-5p, -27a-3p, -96-5p, -129-5p, -130a-3p, 135a-3p, -150a-5p, -182-5p, -200b-3p and -409-3p for further testing of their effects on RSU1. To accomplish this, RSU1 RNA levels were determined at 48 h post miR transfection into MCF10A cells. RNA was isolated and analyzed by quantitative real-time PCR using two sets of RSU1 primers spanning either exon 2–4 (set #1) or exon 5 (set #2). A similar reduction in RSU1 was seen with both primer sets. miRs -7-5p, -18b-5p, -96-5p, -150a-5p, -200b-3p, -409-3p reduced the RSU1 level by 60–80% and miR-182-5p reduced RSU1 RNA by 90% compared to the non-targeting miR control (Figure 1A). A significant reduction in RSU1 RNA followed the transfection of miR-130a-3p and miR-135a-3p as well. The RSU1 protein expression was determined by a Western blot analysis of the miR-transfected MCF10A cells and the results supported the RNA data in many cases. The results in Figure 1B indicated that miR-182-5p as well as miR-7-5p, 200b-3p, 204-3p and 409-3p reduced the levels of the 33kDa RSU1 protein by more than 50% compared to the transfection control and in some cases the reduction was similar to that observed following the depletion of RSU1 with siRNA.

The reciprocal effects of mRNAs on the miRs targeting them can serve as a regulatory mechanism by binding and reducing the level of the miR [22,23,24]. To examine this point, the RNA from MCF10A cells depleted of RSU1 was analyzed in an unbiased miR screen and compared to RNA from negative control cells. Following the reduction in RSU1 RNA with siRNA, the microarray-based analysis was performed. It revealed that miR-130a-3p and miR-204-3p were elevated in MCF10A cells depleted of RSU1 as compared to control cells (Table 2A), suggesting that miR-130a-3p and -204-3p may exhibit a reciprocal relationship with the level of RSU1 RNA. In addition, miRs -744-5p, -221-3p, 1275, -30c-1-3p, -1908-5p, -3656, -3960, -4725-3p and -4792 were elevated in MCF10A cells depleted of RSU1 compared to cells transfected with the negative control siRNA (Table 2A). The effect of the depletion of PINCH1 was examined in the same array-based assay, and the analysis revealed that miR-221-3p and -744-5p were altered by both RSU1 and PINCH1 depletion, suggesting that they may participate in the co-regulation of the binding partners RSU1 and PINCH1 (Table 2B, Supplementary Table S1).

### 2.2. The Expression of miRs Targeting RSU1 in Breast Cancer Cell Lines Varies by Breast Cancer Subtype

Based on the above analyses, the pivotal candidates for the regulation of RSU1 mRNA include miR-182-5p, -18b-5p, -200b-3p, -204-3p, -7-5p, -409-3p, -130a-3p, -221-3p, -744-5p and -106b-5p. Most of these miRs have been reported to play a role in mammary development or breast cancer (Table 3). In addition to the contributions of the individual miRs, the miR-183/-96/-182 cluster is up-regulated in breast cancer [25] and its ectopic expression increases cell proliferation, migration and epithelial mesenchymal transition (EMT) in breast and other cancer cells [26].

**Figure 2:**
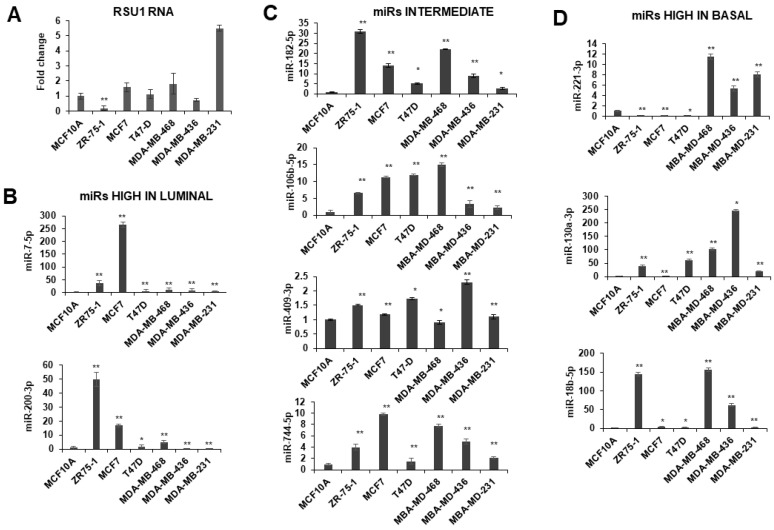
Expression of miRs in breast cancer cell lines. miRs including miR-18b-5p, -130a-3p, -182-5p, -200b-3p, -409-3p, -7-5p, -106-5p, -774-5p and -221-3p were measured in breast cancer cells by TaqMan quantitative PCR. (**A**). RSU1 RNA levels were determined by real-time PCR using primers for exon 3–4. **(B**) The expression of miR-7-5p and -200-3p expression is high in luminal type breast cell lines ZR75-1 and MCF7. (**C**) miR-106-5p, -182-5p, -409-3p and -744-5p are moderate in luminal and basal cells. (**D**) The expression of miR-18b-5p, -130a-3p and -221-3p are higher in basal-like breast cancer cell lines. The expression level of the miRs was normalized to the expression of RNU6B, small nuclear RNA. Values represent the means from triplicate samples ± S.D. * *p* < 0.05 and ** *p* < 0.01 versus control. The MCF10A levels were set to 1.

Initially we examined the RSU1 levels and the expression of miRs targeting RSU1 in human breast cancer cell lines (Figure 2). RSU1 expression is higher in some of the basal type breast cancer cell lines (Figure 2A); PINCH1 RNA is also increased in basal cell lines but there is less variability detected for ILK and parvin (Supplementary Figure S1). The expression of candidate RSU1-targeting miRs in human breast tumor cell lines were coordinated with the hormone receptor status and the breast cancer subtype of the cell lines. As shown in Figure 2, the expression of miR-7-5p and -200-3p is high in estrogen receptor positive (ER+) luminal cells such as ZR75-1 and MCF7 cells (Figure 2B). miR-106-5p and -182-5p are expressed moderately in luminal cells but reduced in some basal cells, while miR-409-3p and -744-5p vary across the subtypes (Figure 2C). The expression of miR-18b-5p, -130a-3p and -221-3p is higher in some basal-like breast cancer cells including MDA-MB-231, MDA-MB-436 and MDA-MB-468 cells (Figure 2D). The differential expression of miRs in various breast cancer phenotypes suggests that these miRs may contribute as regulators of gene expression profiles, differentiating the subtypes of breast cancers.

Based on these analyses of miR levels in breast cancer cell lines and the ability to target RSU1 in MCF10A cells, we focused on a more limited group of miRs for further studies. miR-182-5p and miR-409-5p had been identified with significant physiological changes in tumors and, hence, were included for additional studies. miR-130a and miR-221-3p were included based on the regulation by AKT and the expression in basal breast cancer cells, which are less adhesion-dependent for survival than the luminal lines. In addition, the discovery that miR-130a and miR-221-3p were elevated in RSU1 depleted cells suggested a potential role in RSU1 regulation.

### 2.3. miR-182-5p and miR-409-5p Reduce RSU1 and PINCH1 Thereby Targeting the MKK4-p38-ATF2-PTEN Signaling Pathway in MCF10A Cells

**Figure 3:**
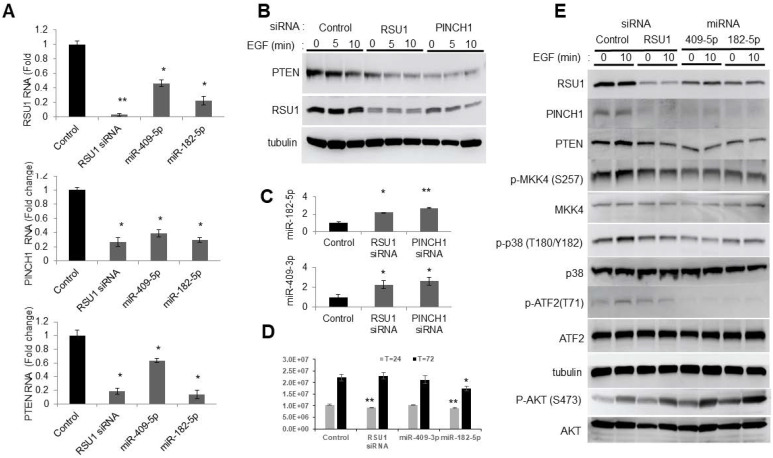
Involvement of the p38 MAPK-PTEN signaling axis following miR-182-5p and miR-409-5p transfection in MCF10A cells. (**A**). The miR-182-5p and miR-409-5p were transfected into MCF10A cells to determine the effect on the levels of RSU1, PINCH1 and PTEN RNA by real-time PCR. Values represent the means from triplicate samples. The data show the means ± S.D., the respective control, *n* = 3. * *p* < 0.05 and ** *p* < 0.01 versus control. Control siRNA levels were set to 1. (**B**). An immunoblot analysis was performed to detect the levels of RSU1 and PTEN expression following siRNA-mediated RSU1 and PINCH1 depletion in MCF10A cells. α-tubulin protein served as the internal control. (**C**). Following siRNA-mediated RSU1 or PINCH1 depletion in MCF10A cells, the expression of miR-182-5p and miR-409-3p was measured by TaqMan real-time PCR. Values represent the means from triplicate samples normalized to the expression of RNU6B, small nuclear RNA. The data represent means ± S.D. * *p* < 0.05 and ** *p* < 0.01 versus control. Control siRNA levels were set to 1. (**D**). The viability of transfected cells was measured at 24 and 72 h post transfection as described in Materials and Methods. The data represent means ± S.D. * *p* < 0.05 and ** *p* < 0.01 versus control. (**E**). The activation of p38 MAPK-PTEN signaling targets was examined following the transfection of miR-182-5p and miR-409-5p in MCF10A cells. An immunoblot analysis was performed with total lysates to detect the levels of RSU1, PINCH1, PTEN, p -MKK4 (S257), p-p38 MAPK (T180/Y182), p-AKT (ser473) and p-ATF2 (T71) expression. The control siRNA and RSU1 siRNA depleted MCF10A cell lysates are included. α-tubulin protein served as an internal control.

Previous studies determined that the level of RSU1 regulates the activation of MKK4-p38-ATF2 and cJun, thereby controlling the transcription of PTEN in MCF10A cells [11]. Hence, the effect of miR-182-5p and miR-409-5p on RSU1 and the signaling pathway leading to ATF2 was examined (Figure 3). After the transfection of miR-409-5p or miR-182-5p into MCF10A cells, the RSU1, PINCH1 and PTEN mRNA expression were determined by quantitative real-time PCR. Both miRs significantly reduced the level of RSU1 RNA and that of its binding partner PINCH1, as well as PTEN (Figure 3A). RSU1 and PINCH1 are binding partners that associate with a high affinity and their levels are co-regulated in numerous cell lines [9,10]. The depletion of either RSU1 or PINCH1 RNA indicates an elevated miR-409-3p and miR-182-5p expression (Figure 3C).

As previously reported, the siRNA-mediated depletion of either RSU1 or PINCH1 reduced PTEN protein expression (Figure 3B) [11]. The transfection of miR-409-5p or miR-182-5p into MCF10A cells targeted RSU1 and PINCH1 (Figure 3E). It also reduced phospho-MKK4, phospho-p38, phospho-ATF2 and PTEN, and increased the phosphorylation of AKT. The changes in the components of the signaling pathwaym resulting from miR-409-5p and miR-182-5p transfection, are comparable to those in cells transfected with RSU1 siRNA. Hence, this suggests that the transfection of miR-182-5p and miR-409-3p reduces the levels of RSU1 and PINCH1 and inhibits MKK4-p38-ATF2-PTEN (Figure 3E). Viability studies indicate that RSU1 depletion transiently inhibits viability, but that miR-182-5p expression inhibited growth for the course of the experiment (Figure 3D).

### 2.4. The Reduction in miR-182-5p Expression in Luminal Breast Cells Increases RSU1 and PINCH1

**Figure 4:**
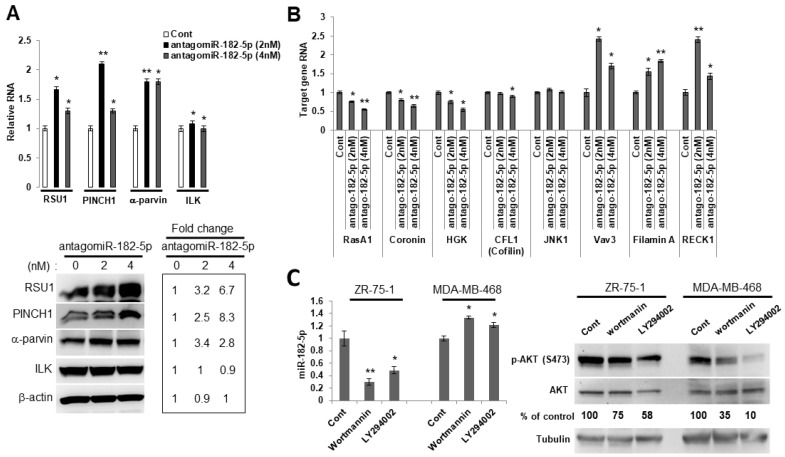
The PI3K/AKT pathway regulates the expression of miR-182-5p in luminal ZR75-1 breast cancer cells. (**A**). RSU1, PINCH1, α-parvin and ILK RNA (upper panel) were determined by real-time PCR and protein levels were determined by Western blot analysis (lower panel) in ZR75-1 cells transfected with the antagomiR-182-5p at either 2 nM or 4 nM. Values represent means from triplicate samples. The data show the means ± S.D.* *p* < 0.05 and ** *p* < 0.01 versus control. Control levels were set to 1. The expression of miR-182-5p was reduced by 95% (2 nM) and 90% (4 nM) by the antagomir-182-5p compared to the non-targeting miR control. (**B**). Candidate target genes in ZR75-1 cells transfected with the antagomiR-182-5p at either 2 nM or 4 nM were analyzed by quantitative real-time PCR. The target genes were normalized to 18S RNA. Values represent the means from triplicate samples and data show the means ± S.D. of the respective control, *n* = 3. * *p* < 0.05 and ** *p* < 0.01 versus control. Control-transfected miR levels were set to 1. (**C**). Luminal ZR75-1 cells or basal-like MDA-MB-468 cells were treated with PI3K inhibitors, wortmannin (100 nM) and LY294002 (15 μM). The inhibition of p-AKT (ser473) was measured by Western blot analysis. The miR-182-5p expression was measured by TaqMan real-time PCR and normalized to the expression of by RNU6B, small nuclear RNA. Values represent the means from triplicate samples. The data show the means ± S.D., the respective control, *n* = 3. * *p* < 0.05 and ** *p* < 0.01 versus control. The control DMSO pretreatment levels were set to 1.

Using another approach to examine the ability of miR-182-5p to regulate RSU1, we chose a cell line with high levels of miR-182-5p expression which could be reduced by an antagomir. The luminal breast cancer cell line, ZR75-1, has a PTEN mutation and constitutively elevated levels of phospho-AKT, and high levels of miR-182-5p are detected in these cells. The transfection of the antagomir to reduce miR-182-5p levels increased RSU1 RNA and protein, as well as PINCH1 and another known miR-182-5p target, α-parvin (Figure 4A), while the expression of ILK was not significantly changed. Furthermore, the expression of additional known targets of miR-182-5p, i.e., VAV3, FLNA (Filamin A) and RECK1, were elevated following a transfection of the antagomiR-182-5p into ZR75-1 cells (Figure 4B). Since the phospho-AKT levels and miR-182-5p expression are high in ZR75-1 cells, we examined the impact of the PI3K/AKT signaling pathway on the expression of miR-182-5p. We treated luminal ZR75-1 and basal MDA-MB-468 cells, which also exhibit constitutive high levels of phospho-AKT, with PI3K inhibitors. Using either wortmannin or LY294002 to inhibit the activation of AKT, the miR-182-5p expression was suppressed in the luminal ZR75-1, but not in basal cell line MDA-MB-468 (Figure 4C), suggesting that AKT signaling regulates miR-182-5p in ZR75-1 cells but not in the basal MDA-MB-468 cells.

### 2.5. miR-221-3p and miR-130a-3p Target RSU1 and Other RNAs in MCF7 Luminal Breast Cancer Cells

The finding that RSU1 depletion elevated miR-221 was relevant because miR-221 is known to target RNAs and cell functions linked to IPP and RSU1. miR-221 and miR-222 interfere with CDKN1B/p27^KIP1^, CDKN1C/p57, PTEN and TIMP3 expression and TRAIL-induced caspase machinery [49,50]. miR-221 and -222 levels are increased by AKT signaling from receptor tyrosine kinase activation and other pathways, and miR-221 and -222 increase the activation of JNK, cJun and AP1, thereby enhancing cellular invasion and migration [51,52,53]. Previous reports determined that miR-130a modulates miR-221 and -222 expression through AP1 [43,49] and that miR-130a targets PTEN via the PI3K and AKT, Wnt/β-catenin and NF-kB signaling pathways [43]. Because miR-130a-3p targets RSU1, and both miR-221-3p and miR-130a-3p are elevated by RSU1 depletion (Table 2), we investigated their role in RSU1 regulation, particularly the involvement of P13K/AKT and PTEN signaling pathways.

**Figure 5:**
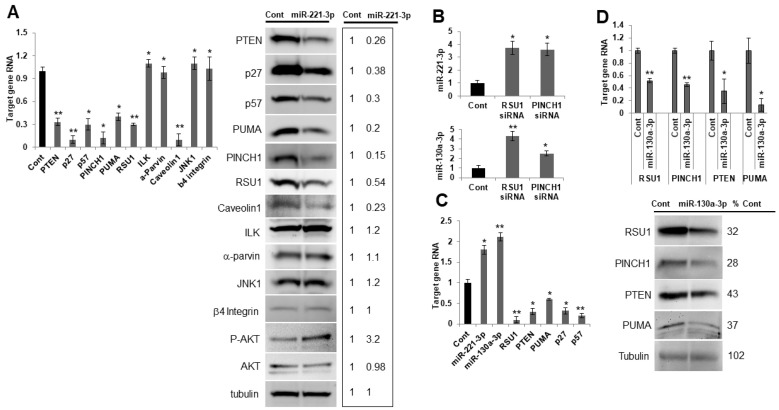
miR-221-3p and miR-130-3p target RSU1 and other candidate genes in MCF7 luminal breast cancer cells. (**A**) MCF7 cells were transfected with the mimic miR-221-3p or control miR. The RNA level of targets was measured by real-time PCR. RNAs include PTEN, p27, p57, PINCH1, PUMA, ILK, α-parvin, caveolin1, JNK1 and β4 integrin (left panel) and the protein levels were analyzed by Western blot analysis (right panel), including PTEN, p27, p57, PINCH1, PUMA, ILK, α-parvin, caveolin1, p-AKT, JNK1 and β4 integrin. RSU1 expression was used as a positive control. RNA values show the means from triplicate samples ± S.D. * *p* < 0.05 and ** *p* < 0.01 versus control. Negative control miR-transfected levels were set to 1. Note that the expression of miR-221-3p was increased >20-fold by mimic miR-221-3p compared to the non-targeting miR control in MCF7 cells. (**B**). The expression of miR-221-3p and miR-130a-3p was measured by real-time PCR in MCF7 cells depleted of RSU1 or PINCH1. The expression level of miRs was normalized to the expression of RNU6B, small nuclear RNA. Values represent the means from triplicate samples ± S.D. * *p* < 0.05 and ** *p* < 0.01 versus control. (**C**). Following the depletion of RSU1 in MCF7 cells, the expression of miR-221-3p and -130a-3p was measured by TaqMan real-time PCR and the target gene RNA was measured by quantitative real-time PCR. Values represent the means from triplicate samples. The data show the means ± S.D. * *p* < 0.05 and ** *p* < 0.01 versus control. Control siRNA levels were set to 1. (**D**) RSU1, PTEN, PINCH1, and PUMA RNA levels were determined by real-time PCR in MCF7 cells transfected with miR-130a-3p. Data were normalized to 18S. Values represent the means ± S.D. * *p* < 0.05 and ** *p* < 0.01 versus control. Negative control siRNA levels were set to 1. The changes in protein expression were examined by Western blot analysis. Results from the quantitation of the blots were normalized to tubulin and are expressed as a percentage of the negative control-transfected cells.

miR-221-3p and miR-130a-3p were transfected into MCF7 cells which express a low endogenous level of the miRs, as seen in Figure 2D, and where the effect of the elevated levels would be detectable. The increase in miR-221-3p reduced the levels of RSU1, PINCH, PTEN, p27^KIP^, p57, PUMA, and caveolin 1 RNA and protein (Figure 5A), while no change was detected in JNK1 or β4 integrin. The siRNA-mediated depletion of RSU1 and PINCH1 resulted in a reciprocal elevation of miR-221-3p and -130a-3p in MCF7 cells (Figure 5B). Accordingly, the depletion of RSU1 and elevation of miR-221-3p and miR-130a-3p decreased PTEN, PUMA, p27^KIP^ and p57 RNA (Figure 5C). The RSU1, PINCH1, PTEN and PUMA RNA levels were inhibited by overexpressed miR-130a-3p (Figure 5D).

### 2.6. miR221-3p and miR-130a-3p Are not Regulated by p38 MAPK

**Figure 6:**
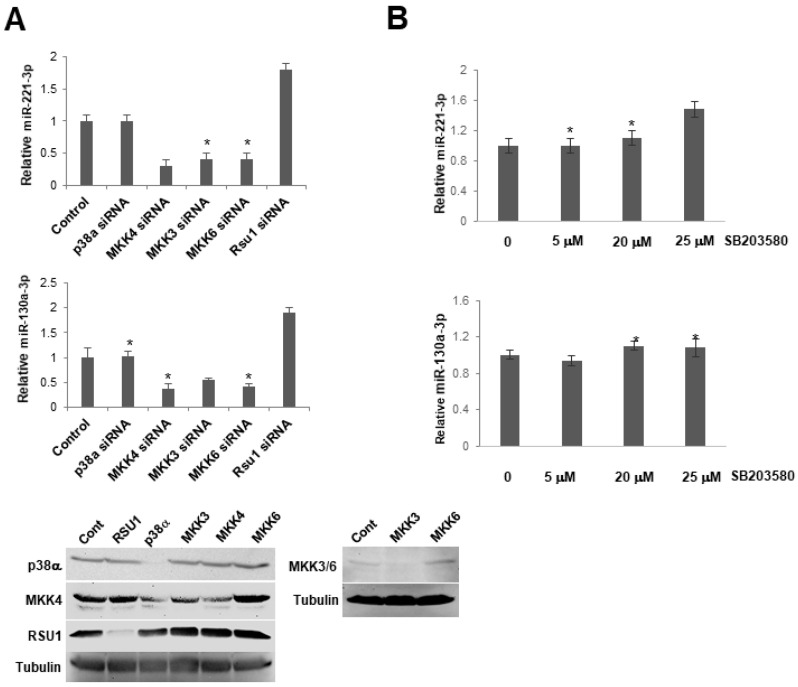
p38 MAPK activation does not regulate the expression of miR-130a-3p and miR-221-3p in MCF7 cells. (**A**). The expression level of miR-221-3p and -103a-3p was measured by TaqMan real-time PCR following the transfection of siRNAs for p38α MAPK, MKK4, MKK3, MKK6 into MCF7 cells. Values represent the means from triplicate samples ± S.D. * *p* < 0.05 versus control and control siRNA levels were set to 1. Western blot analysis shows the depletion of p38, MKK4, MKK3, 6 by siRNA in these cells. (**B**). MCF7 cells were pretreated with a p38 MAPK inhibitor, SB203580, for 1 h. The expression level of miR-221-3p and -103a-3p was measured by TaqMan real-time PCR. The expression level of miRs was normalized to the expression of by RNU6B, small nuclear RNA. Values represent means ± S.D. * *p* < 0.05 versus control. Control DMSO pretreatment levels were set to 1.

RSU1 depletion resulted in elevated miR-221-3p and -130a-3p. Both miRs can be induced by AKT signaling, and RSU1 depletion can elevate AKT phosphorylation by the decreasing MKK4-p38-ATF2-PTEN. To determine the involvement of p38 MAPK or its upstream activators, MKK3/6 or MKK4, in miR-130a and miR-221-3p expression, p38α, MKK3/6 and MKK4 siRNA were transfected into MCF7 cells (Figure 6A). The results indicate that depleting components of these pathways did not have the same effect as the RSU1 depletion. Exposing MCF7 cells to the p38 inhibitor SB203580 did not significantly affect the expression of miR-221-3p and -130a-3p (Figure 6B). Hence, if the elevation of miR-221-3p and -130a-3p by RSU1 depletion is a result of the loss of PTEN and an elevated AKT signal, it does not require the inhibition of MKK4, MKK3/6 or p38 function.

**Figure 7:**
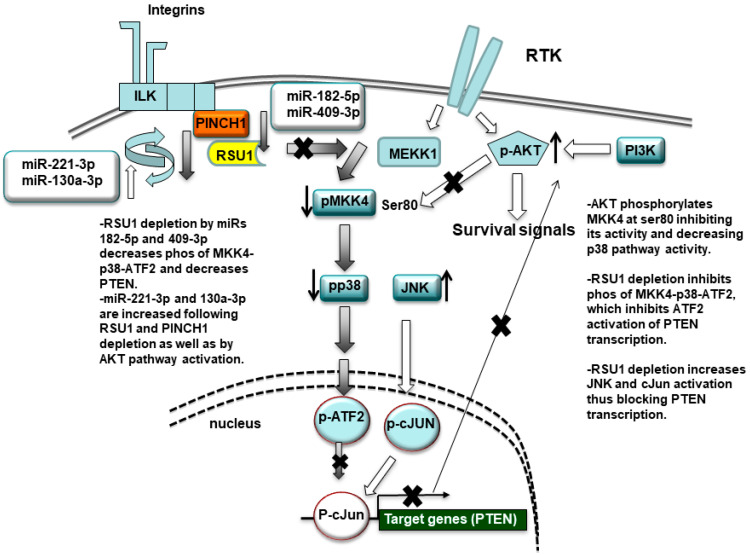
Schematic summary of the miR-targeting of RSU1. RSU1 depletion by miRs-182-5p and -409-3p decreases the phosphorylation of MKK4-p38-ATF signaling. miRs-221-3p and -130a-5p target RSU1 and PINCH1 and are increased by RSU1 and PINCH1 depletion. RSU1 plays an important role for the transcriptional regulation of the PTEN gene through the regulation of the promotor binding factors ATF2 or cJun, which are dependent on upstream activation by p38 MAPK and JNK, respectively. The active AKT phosphorylates MKK4 at ser80 and suppresses its activity and downstream p38 MAPK signaling.

In summary, we conclude that RSU1 and members of the IPP adhesion complex are targeted by miRs linked to migration, metastasis as well as cell cycle progression. The impact of the miRs on RSU1 was examined in MCF10A mammary epithelial cells and two luminal breast cancer cell lines. As illustrated in Figure 7, the reduction in RSU1 by miR-182-5p and -409-3p results in similar changes in PTEN as those previously observed upon RSU1 depletion. This may constitute a novel mechanism for the regulation of the PTEN/PI3K/AKT signaling pathway by miR-182-5p and -409-3p, via the targeting of RSU1 and the IPP cell adhesion complex in mammary epithelial and breast cancer cells. In addition, the targeting of RSU1 and PINCH1 by miR-221-3p, and the reciprocal increase in miR-221-3p and miR-130a by RSU1 reduction, suggests an additional feedback mechanism to regulate miR-221 and miR-130 and their targets. This may occur as a result of a decrease in PTEN following RSU1 depletion.

## 3. Discussion

Breast cancer initiation and progression is promoted by the stromal environment, and the induced changes that drive tumorigenesis include epigenetic alternations [54,55]. Changes in RNA stability and translation resulting from the miR transcription in tumor cells, or from miR-containing exosomes transferred between tumor, stromal, and other cells in the microenvironment, can drive breast tumor initiation and metastasis [56,57]. The miRs produced by local stromal cells can be detected in breast tumor tissue [58]. Therefore, the observation that the exosome-mediated miR-induced depletion of RSU1 correlates with the tumor initiation and metastasis in prostate cancer, and that RSU1 expression is altered between normal and tumor tissue in breast tumors [59], supports a role for miR regulation of the RSU1 function in breast tumor development and/or progression.

This study was designed to identify miRs targeting RSU1, or members of the IPP complex, and their activity in normal breast and luminal breast cancer cell lines. We show that RSU1 is a direct target of miR regulation in both normal and luminal breast tumor cell lines. Two of the miRs targeting RSU1, miR-409-3p/-5p and -182-5p, have been linked to altered epithelial cell growth early in tumor initiation and the development of tumor metastases [15,16,17]. In addition, several of the miRs that target RSU1 regulate adhesion, spreading and migration, activities which require RSU1 and the IPP complex.

The data from this study show that RSU1 RNAs and proteins are significantly decreased by miR-409-3p/-5p or miR-182-5p. Furthermore, the data reveal that the depletion of RSU1 also increased the expression of miR-409-3p/-5p and miR-182-5p, suggesting these miRs are also targets of RSU1 or RSU1-dependent signaling. It is known that miR-409-5p activates the AKT pathway [40], and via the depletion of RSU1, promotes exosome-mediated prostate tumors. miR-409-3p is a tumor suppressor which is decreased in breast tumors, and its expression targets AKT in breast cell lines. In contrast, miR-182-5p expression is significantly higher in tumors, including breast cancer [25,60], compared to normal tissue [61,62]. In addition to reducing RSU1, miR-182-5p promotes tumor cell proliferation, invasion and migration by targeting FOXF2, RECK and MTSS1 [61], and the suppression of MTSS1 (MIM) by miR-182 activates RhoA and promotes breast cancer metastasis [63]. RhoA regulates the cell shape and polarity and it enables cell migration by promoting the detachment and dissolution of focal adhesions [64]. Similarly, our group and others have demonstrated a requirement for PINCH1–RSU1 interaction to promote focal adhesion formation, cell adhesion and migration in mammary epithelial cells [8,59,65,66]. Hence, the loss of RSU1, and RSU1–PINCH1 binding, disrupted the connection of the integrins to the actin cytoskeleton required for adhesion and migration. Therefore, among other outcomes, miR-182-5p can inhibit adhesion, spreading and migration by targeting RSU1 and disrupting the IPP complex.

Previous work has determined that the expression of RSU1 inhibits the phosphorylation of AKT (S473) in breast cancer cell lines, and the depletion of RSU1 increases PI3K/AKT signaling [11]. This occurs via the transcriptional control of the tumor suppressor PTEN by a RSU1-MKK4-p38-ATF2-induced transcriptional regulatory mechanism. Following the depletion of RSU1, a decreased ATF2 activation resulted in reduced ATF2 binding in the PTEN promoter region. This was accompanied by an elevated JNK activation and increased cJun binding to a negative regulatory site in the PTEN promoter, thus reducing transcriptional activation. Hence, our current study examined the effect of miRs 182-5p and miR-409-3p on PTEN expression. The reduction in RSU1 by miR-182-5p and miR-409-3p in MCF10A mammary epithelial cells inhibited PTEN and the same MKK4-p38-ATF2 signaling pathway to PTEN that we previously identified. This finding may be clinically useful since the phosphorylation of ATF2 within the activation domain is a key determinant of sensitivity to tamoxifen in luminal breast cancer [67].

The ZR-75-1 luminal breast cancer cells have a PTEN mutation and elevated P-AKT; these cells also express high levels of miR-182-5p, thus providing a model for examining the effect of miR-182-5p depletion on RSU1 and IPP RNAs and proteins. As hypothesized, the reduction in miR-182-5p by its antagomir elevated the expression of RSU1, PINCH1 and parvin. Moreover, the results in Figure 4 demonstrate that the expression of miR-182-5p is dependent on the PI3K/AKT signaling pathway in ZR75-1 cells (Figure 4C), suggesting that the expression of miR-182-5p may be correlated with PTEN and the AKT regulation in the luminal breast tumor cell lines.

The other RSU1-targeting miRs explored in this study, miR-221 and -130a-3p, are well established as regulators of cell signaling. RTK signaling increases the miR-221/-222 levels via the AKT activation coincident with EMT and the activation of transcription factors cJun and AP1, thereby enhancing cell invasion and migration [51,68]. miR-221/222 are elevated in metastatic breast cancer and increased levels are prognostic in lymph node positive and HER2 positive tumors [69]. miR-221 expression also targeted RSU1, as well as causing a reduction in PTEN, and increased P-AKT. The miRs -221/-222 also interfere with p57, p27^KIP1^, PTEN and TIMP3 expression, as well as TRAIL-induced caspase machinery [50]. Therefore, the effects of miR-221-3p on PTEN may be direct or not require RSU1 regulation. Furthermore, miR-130a can modulate the miR-221 and miR-222 expression levels through AP1/cJun [49]. Taken together, miR-221/-222 and -130a, which target RSU1 and other signaling proteins, are closely correlated with the levels of PI3K/AKT and PTEN signaling pathways [70]. A disruption of the control of AKT signaling is common to the expression of tumor-associated miRs. The miRs that target the tumor-suppressive protein phosphatases including PTEN, PHLPP2 and INPP4B, which elevate the PI3K pathway activity in luminal breast cancer [71,72].

Tamoxifen resistance can result from alterations in PI3K/AKT signaling pathway in luminal breast cancer cells [73]. An elevated AKT increases miR-221-3p which targets the estrogen receptor RNA thereby reducing the number of estrogen receptors and the sensitivity of cells to tamoxifen [74]. Although the reduction in RSU1 increases AKT and miR-221, the transfection of miR-221-3p into MCF7 cells did not alter the level of ERα RNA (data not shown).

In conclusion, our data propose a novel mechanism for the regulation of the PTEN/PI3K/AKT signaling pathway in mammary epithelial and breast cancer cells by miRs targeting RSU1 and PINCH1. Dysregulation of miRs is associated with the failure of chemotherapy, radiation therapy, anti-endocrine therapies, and other targeted therapies in breast cancer [75,76]. Therefore, the study of candidate miR expression may yield a better understanding of the processes leading to metastasis. Additionally, the detection of miRs in tumor tissue and the circulation may provide useful biomarkers for the diagnosis and prognosis of breast cancer.

## 4. Materials and Methods

### 4.1. Cell Lines and Culture

MCF10A mammary epithelial cells were maintained in a DMEM-F12 medium as described [8]. Human breast cancer cell lines, ZR75-1, MCF7, T47D, MDA-MB-231, MDA-MB-436 and MDA-MB-468, were used as described previously [10,77]. Cells were obtained from American Type Culture Collection. The viability of cells was measured using The CellTiter-GloR 2.0 Assay (Promega, Madison, WI, USA). The assay determined the number of metabolically active and viable cells in culture by quantitating the amount of ATP present, where the amount of ATP was directly proportional to the number of cells present in culture. Cells were seeded in 96 well plates at 10^4^ per well in quadruplicate for each time point and were analyzed at the times indicated as recommended by the manufacturer. The luminescence was measured on a GloMax Discover luminometer (GM3000 Promega).

### 4.2. Reagents

EGF, SB203580, wortmannin and LY294002 were purchased from Sigma-Aldrich (St. Louis, MO, USA).

### 4.3. siRNA-Induced Gene Silencing

38α (MapK14), MKK3 (Map2K3_5), MKK6 (Map2K6_5) and MKK4 (Map2K4_5) siRNA were purchased from Qiagen (Valencia, CA, USA). The control siRNA is AllStars negative control siRNA from Qiagen (Valencia, CA, USA). siRNAs for RSU1, PINCH1, parvin and ILK were from Thermo Fischer Scientific.

### 4.4. miR Target Predictions

We used three commonly used tools for predicting miRs: miRanda (August 2010 release), TargetScan (released 7.1, June 2016), and MicroT-CDS (version 5.0, updated July 2012). For miRanda [78], we used human target site predictions with good mirSVR scores and conserved miR. The scale values ≤ 0.1 predicted a higher possibility of RSU1-targeting. For TargetScan [79], Context+++ score is to be most negative and the Context score and *p*_CT_ (probability of conserved targeting) were used to evaluate the probability of a miR to bind 3′-UTR of RSU1. For microT-CDS [80], we used the gene symbol “RSU1”, species “Homo sapiens”, gene ID “ENSG00000148484”. The results were evaluated using the miTG score; 0.7–1 or 0–1 scores predict a higher possibility of targeting in the case of the miTG score.

### 4.5. MicroRNA Array Analysis

Total RNA was isolated with TriPure isolation reagent (Roche Applied Science, Indianapolis, IN, USA) according to the manufacturer’s instructions. A total of 5 micrograms of RNA for each sample was sent to perform a microRNA microarray expression analysis using HmiOA7.1 Human miR OneArray Chip by Phalanx Biotech group. It contains miR-specific probes and a proprietary spacer designed to enhance the hybridization sensitivity to miRs. According to the manufacturer’s instructions, miR probes are each spotted three times.

### 4.6. Western Blotting and Antibodies

Western blotting assays were performed as described previously [11]. In brief, protein lysates for the Western blot analysis were prepared in the RIPA buffer (50 mM Tris–HCL, pH 7.5, 150 mM NaCl, 1% NP-40, 0.5% Doc, 0.1% SDS with protease inhibitors) or a high salt lysis buffer (400 mM NaCl, 10 mM HEPES, pH 7.5, 1.5 mM MgCl_2_, 0.1 mM EDTA, 5% glycerol, 1 mM DTT with protease inhibitors). The protein concentration was detected by a BCA protein assay (thermo scientific) and equal amount of proteins were loaded into a 10% or 12% Novex SDS-PAGE Wedgewell gel. After gels were transferred to PVDF membranes, the membranes were blocked in 2% skim milk for 1 h at room temperature, then incubated with the primary antibodies overnight at 4°. The membranes were incubated in a horseradish peroxidase (HRP)-conjugated secondary antibody (Santa Cruz Biotechnology, Dallas, TX, USA) for 1 h at room temperature and detected by ECL chemiluminescence (Millipore, Burlington, MA, USA). The LAS-4000 Luminescent image analyzer (Fuji medical system, Roselle, IL, USA) was used for analyzing the protein bands. The same membranes were reblotted for normalization and as a control for the amounts of protein loaded with monoclonal anti-α-tubulin and β-actin antibodies (Sigma, Saint Louis, MO, USA). The anti-p-p38 (T180/Y182, #4511), p-MKK4/SEK1 (S257, #4514), p-ATF2 (T71, #5112), PTEN (#9188), p27 (#3698), and PUMA (#4976) antibodies were purchased from Cell Signaling Technology (Beverly, MA, USA). The anti-α-tubulin (sc-8035), MKK4 (sc-166168), MKK3,6 (sc-133230), JNK1 (sc-571), ILK (sc-13075), ATF2 (sc-187), P-AKT1,2,3 (ser473-4 (sc-514032)) and p57 (sc-1040) were also obtained from Santa Cruz Biotechnology (Santa Cruz, CA, USA). The anti-PINCH1 (#612710), caveolin1 (#610406) and integrin β4 (#610467) antibodies used were from BD Biosciences (San Jose, CA, USA); p38α/β2 antibodies (#05-454), AKT (#05-591), were from Millipore (Billerica, MA, USA). Parvin antibodies were from Sigma (Sigma, Saint Louis, MO, USA). The anti-RSU1 antibodies were used as described previously [8].

### 4.7. RNA Isolation and Quantitative Real-Time RT-PCR

Total RNA was isolated with TriPure from the manufacturer’s protocols of Roche (Indianapolis, IN, USA) and cDNA synthesis was performed by a GeneAmp RNA PCR Core Kit (Applied Biosystems, Foster City, CA, USA). Primer sequences for real-time RT-PCR were used as described in Supplemental Table S1 with FastStart Universal SYBR Green Master (Roche), and was analyzed on an ABI 7500 real-time PCR machine (Applied Biosystems, Foster City, CA, USA). The primer was set for 18S ribosomal RNA: 5′-GGATCCATTGGAGGGCAAGT-3′ (forward) and 5′-AATATACGCTATTGGAGCTGGAATTAC-3′ (reverse) was also amplified to normalize the results. Representative results are shown as triplicate or quadruplicate samples normalized with the control samples. All primer sequences are listed in Supplementary Table S1.

### 4.8. miR Isolation and miR Quantitative Real-Time RT-PCR

The mirVana miRNA Isolation Kit was used for isolating small RNAs cells according to the manufacturer’s protocols (AM1561, Thermo fisher scientific). A 10 ng of small RNA was transcribed into cDNA using a TaqMan MicroRNA Reverse Transcription kit (#4366596, Thermo Fisher Scientific, Waltham, MA, USA). The expression of all miRs was quantified and validated by using a TaqMan MicroRNA Assay kit (cat. no. 4,427,975, Thermo Fisher Scientific) following the manufacturer’s protocol, which included primers for has-miR-18b-5p (assay ID: #002717), -130a-3p (#000454), -182-5p (#002334), -200b-3p (#002300), -409-3p (#002332), -7-5p (#000268), -106b-5p (#000442), -744-5p (#002324) and -221-3p (#000524). Real-time PCR reactions were performed in StepOnePlus (Applied Biosystems). The qPCR was performed using a 7500 Real-Time PCR system with TaqMan Universal Master Mix II, no UNG (Thermo Fisher Scientific, #4440043) with triplicate samples. The PCR conditions were as follows: 10 min at 95 °C before 40 thermal cycles, each was 15 s at 95 °C and 1 min at 60 °C. The expression level of miRs was normalized to the expression of RNU6B, small nuclear RNA (#001093), expression and were calculated using the 2^−ΔΔCt^ method with the triplicate sample.

### 4.9. Statistical Analysis

Representative results of the triplicate or quadruplicate realtime PCR samples were normalized with the control samples 18S or RNU6B. The means and standard deviation were calculated (S.D.). The student’s T test was used for comparison between the means of respective groups. * *p* < 0.05 and ** *p* < 0.01 versus control are represented in the figures.

### 4.10. Mimic and Antagomir Transfection

MirVana mimics for miR-18b-5p (MC10466), -27a-3p (MC10939), -96-5p (MC10422), -130a-3p (MC10506), -135a-3p (MC23887), -150-5p (MC10070), -182-5p (MC12369; antagomiR: MH12369), -200b-3p (MC10492), -409-3p (MC12446), -409-5p (MC13028), -7-5p (MC11755), -204-3p (MC19554), -221-3p (MC10337) and -129-5p (MC10195), along with a miR-negative control, were transfected into the cells using the Lipofectamine RNAiMAX transfection reagent (Thermo Fisher) with the reverse transfection method as described in the manufacturer’s instructions.

## Figures and Tables

**Table 1:** miRs predicted to target RSU1 identified using common algorithms.

miRs.	miTG Score	Pct	Context+++ Score	mirSVR Score
7a/b-5p		< 0.1	−0.38	−0.9329
18a/b-5p	0.74	< 0.1	−0.34	
22-3p		0.55	−0.32	−0.4965
27a-3p	0.71			−0.5595
33a/b-5p		0.18	−0.13	−0.1007
96/507/1271-5p	0.72	0.74	−0.03	−0.0274
106a-5p	0.76	N/A	−0.12	−0.0298
129-5p	0.76	< 0.1	−0.18	−0.5599
130a/301/454/721-3p		< 0.1	−0.02	
132/212-3p		N/A	−0.12	−0.1108
135a-5p		< 0.1	−0.15	−0.2668
150/5127-5p	0.75	< 0.1	−0.12	
182-5p		0.74	−0.10	−0.0277
183		< 0.1	−0.18	−0.3810
186-5p	0.82	N/A	−0.06	−0.2116
188-3p	0.8	N/A	−0.07	
200bc/429/548-3p	0.97	< 0.1	−0.06	−0.4047
204-3p/4646-5p		N/A	−0.13	
204/211-5p		< 0.1	−0.13	−0.0982
320a		N/A	−0.03	−0.0100
384	0.87	N/A	−0.16	−0.4510
409-3/5p		N/A	−0.27	
495-3p	0.73	N/A	−0.08	−0.8570

miRs predicted to target RSU1 RNA were identified using three common algorithms. The gene “RSU1”, species “Homo sapiens”, gene ID “ENSG00000148484” was used to identify miRs predicted to bind RSU1 RNA by employing three common algorithms including MicroT-CDS, TargetScan and miRanda. In the MicroT-CDS analysis, a miTG score of 0.7–1, from a 0–1 scale, predicts a higher likelihood of targeting with greater the confidence as the score is closer to 1.0. Pct (Probability of Conserved Targeting) from TargetScan. The higher the score between 0 and 1, the greater the conservation and the greater mRNA destabilization expected. Using Context+++ score (value range from 1 to −1) from TargetScan analysis a more negative score predicts greater likelihood of miR binding. The mirSVR score values <0.1 predict higher possibility of RSU1-targeting for miRanda. Hence, the more negative the score, the greater the probability of binding.

**Table:** **A.** Array based analysis reveals miRs elevated following RSU1 depletion in MCF10A cells.

	Array Analysis (*RSU1 Depletion*)	
miR	*T/C*	*p* Value
130a-3p	1.853467	0.000166817
744-5p	2.240783	0.00000251
221-3p	1.900005	0.0000982
1275	1.748783	0.00000628
30c-1-3p	1.747629	0.0000347
1908-5p	1.755459	0.0000262
3656	1.940992	0.00000416
3960	2.347732	0.000449102
4725-3p	2.080193	0.000121922
4792	1.788032	0.000000192
204-3p	2.399785	0.000000147

**Table:** **B.** Validation of miR expression change following in RSU1 or PINCH1 depletion.

	**Quantitative Taqman RT-PCR (*RSU1 Depletion*)**		**Quantitative Taqman RT-PCR (*PINCH1 Depletion*)**	
**miR**	***T/C***	***p* Value**	***T/C***	***p* Value**
221-3p	4.74	0.0062378	4.28	0.01376867
744-5p	2.36	0.0469889	2.26	0.00987941
**Quantitative Taqman RT-PCR (*RSU1 Depletion)***				
**miR**	***T/C***		***p* Value**	
1275	1.2		0.0692250893	
30c-1-3p	1.4		0.002352315	
1908-5p	1.2		0.0094784933	
4725-3p	1.5		0.0201897997	

(**A**) miR array-based analysis reveals miRs elevated during RSU1 depletion in MCF10A cells. Note that these are 11 target miRs among 14 up-regulated miRs in RSU1 knock-down MCF10A cells with a fold induction > 1.74. The human microRNA microarray assay was performed by a HmiOA7.1 Human miRNA OneArray Chip. (**B**) The validation of the expression changes for miRs targeting RSU1 and/or PINCH1 RNA following the depletion of RSU1 or PINCH1 in MCF10A cells. Experimental validation of the representative miR expression by TaqMan quantitative by real-time PCR. Note that the mean of C/T were determined for miR-221-3p, -744-5p, -1275, -30c-1-3p, -1908-5p and -4725-3p. The expression level of the miRs was normalized to the expression of RNU6B, small nuclear RNA. Values represent the means from triplicate samples ± S.D. The *p* value is for versus control.

**Table 3:** Association of RSU1 targeting miRs with breast development and disease.

microRNA	Breast Association	Reference
miR-182-5p	-Increased in breast tumor versus normal tissue	[27]
	-Associated with veliparib resistance	[28]
	-Independent predictor of benefit from endocrine therapy	[29]
miR-200b-3p	-Independent predictor of benefit from endocrine therapy	[30,31,32,33]
	-Regulates EMT in breast through ZEB1/2	
miR-204-3p	-Part of a signature to predict recurrence free survival	[34,35]
	-Targets TGFb2 and ANGPT1 to inhibit angiogenesis and vascularization	[28]
	-Targets FOXA1; inhibits proliferation and migration	[36]
	-Targets IL11 and TGF beta signaling	[37]
miR-7-5p	-Significant lower level of miR-7 in metastatic breast cancer tissues than normal breast tissue and regulation of EMT process by E-cadherin and vimentin expression	[35]
	-Inhibition of EGFR and PKB for growth and metastasis of breast cancer cells	[38]
miR-409-3p	-miR-409-3p inhibited cellular growth, migration and invasion by targeting ZEB1	[39]
	-miR-409-3p induced downregulation of Akt1 protein through binding to 3’UTR	[40]
miR-130a-3p	- Overexpression of miR-130a inhibited migration and invasion in MDA-MB-231 by reduction of FOSL1 and upregulation of ZO1	[41]
	-miR-130a-3p inhibits migration and invasion by regulating RAB5B	[42]
	-miR-130a overexpression in human breast cancer cells promoted Akt phosphorylation, cell survival, and tumor growth by repression of PTEN	[43]
miR-221-3p	-PAK1-mediated phosphorylation of serine 305 (S305) of ERα by miR-221-3p targeting the 3’ UTR of PAK1	[44]
	-ADAMTS6 inhibits tumor development by regulating the ERK pathway via binding of miR-221-3p	[45]
miR-744-5p	-miR-744-5p suppresses the MCF7 cancer cell growth by reducing proto-oncogene EF1A2	[46]
miR-106-5p	- MicroRNA-106b targets FUT6 to promote migration, invasion, and proliferation in human breast cancer	[47]
	-miR-106b-5p was significantly upregulated in BRCA cells and contributes to the lung metastasis of breast cancer by inhibition CNN1 and activating Rho/ROCK1	[48]

## Supplementary Materials

Supplementary Materials can be found at https://www.mdpi.com/1422-0067/21/15/5458/s1.

## Abbreviations

RSU1	Ras suppressor protein
PINCH1	LIMS1
ILK	Integrin linked kinase
PTEN	phosphatase and tensin homolog
AKT	Protein kinase B
MiR	microRNA
