# Supplementary material for: MicroRNA-Dependent Targeting of RSU1 and the IPP Adhesion Complex Regulates the PTEN/PI3K/AKT Signaling Pathway in Breast Cancer Cell Lines
Source: Int J Mol Sci. 2020 Jul 30;21(15):5458. doi: 10.3390/ijms21155458 (PMC7432699; doi:10.3390/ijms21155458)
Supplement: Supplementary file 1 [file ijms-21-05458-s001.zip › Supplemental figures/Supplemental Table 1. primer sequences.pdf]

Supplemental Table 1. Primer sequences used for quantitative realtime PCR

Gene symbol& size	Region	Sequences
**RSU1**	*RSU1_F (Exon 2/3/4)*	5'-GGATGTCAACGGCCTCTTT-3'
(76 bp)	*RSU1_R (Exon 2/3/4)*	5'-TGGCACCATTGTTAGCTTGT-3'
	RSU1_F (Exon 5)	5'-GCATGAACAGGCTGAACACT-3'
(71 bp)	*RSU1_R (Exon 5)*	5'-CGTCAAGTCCAGAACCTCAA-3'
**PINCH1/LIMS1**	*PINCH1_F (Exon 2/3)*	5'-CCGCTGAGAAGATCGTGAAC-3'
(150 bp)	*PINCH1_R (Exon 2/3)*	5'-GGGCAAAGAGCATCTGAAAG-3'
**ILK**	*ILK_F*	5'-GCCCTTGTCAGCATCTGTAA-3'
(143 bp)	*ILK_R*	5'-CCCTTCCAGAATGTGTCCTT-3'
**α-Parvin**	*α-Parvin_F*	5'-AGCATCAAGTGGAATGTGGA-3'
(88 bp)	*α-Parvin_R*	5'-CGCGGAAATACTGAGACAGA-3'
**PTEN**	*PTEN_F (exon 1)*	5'-GATGTGGCGGGACTCTTTAT-3'
(121 bp)	*PTEN_R (exon 1)*	5'-AGCGGCTCAACTCTCAAACT-3'
**p27**	*p27_F*	5'-GTGATCACTCCAGGTAGTT-3'
(484 bp)	*p27_R*	5'-CAAATGCACAAAACATGCCA-3'
**p57**	*p57_F*	5'-CTCCGCAGCACATCCACGAT-3'
(66 bp)	*p57_F*	5'-GGTGCGCACTAGTACTGGGA-3'
**PUMA**	*PUMA_F*	5'-GTTCCAGCTGCAGGGGTG-3'
(62 bp)	*PUMA_R*	5'-CAGAGTGAAGGAGCACCGAG-3'
**RasA1**	*RasA1_F*	5'-TGGACGAAGGTGACTCTCTG-3'
(127 bp)	*RasA1_R*	5'-AGGCGTTCTTCTGCTATCGT-3'
**RECK1**	*RECK1_F*	5'-CCATCTGGAGATCCCTGTCT-3'
(100 bp)	*RECK1_R*	5'-GCACCTGGATTAGTGTCCCT-3'
**HGK**	*HGK_F*	5'-CTGGTCACTTGGATGGTGTG-3'
(199 bp)	*HGK_R*	5'-TGAAGACTCTGGAGGCCAGT-3'
**VAV3**	*Vav3_F*	5'-TCACTCGAGTCACATCCTGC-3'
(103 bp)	*Vav3_R*	5'-CATTCTTTGTGTGCTCTCGC-3'
**Coronin**	*Coronin_F*	5'-ACCCAACCTCATCTCCTTG-3'
(63 bp)	*Coronin_R*	5'-CCACCTTGAGATCCCTGTTT-3'
**FLNA**	*FilaminA_F*	5'-GCATCAAACTGGTGTCCATC-3'
(85 bp)	*FilaminA_R*	5'-AGGATCAGGGTCCAGATGAG-3'
**Caveolin1**	*Caveolin1_F*	5'-CACATCTGGGCAGTTGTACC-3'
(94 bp)	*Caveolin1_R*	5'-CACAGACGGTGTGGACGTAG-3'
**JNK1**	*JNK1_F*	5'-TTGGAACACCATGTCCTGAA-3'
(241 bp)	*JNK1_R*	5'-ATGTACGGGTGTTGGAGAGC-3'
**PAK1**	*PAK1_F*	5'-CCCATTTCACCTACTGAAAATAACA-3'
(131 bp)	*PAK1_R*	5'-CCCACACTCACTATGCTTCG-3'
**β4 integrin**	*β4 integrin_F*	5'-CTGTACCCGTATTGCGACT-3'
(220 bp)	*β4 integrin_R*	5'-AGGCCATAGCAGACCTCGTA-3'
**CFL1 (Cofilin)**	*CFL1 (Cofilin)_F*	5'-GTGCCCTCTCCTTTTCGTTT-3'
(75 bp)	*CFL1 (Cofilin)_R*	5'-TTGAACACCTTGATGACACCAT-3'
