# Supplementary material for: MicroRNA-Dependent Targeting of RSU1 and the IPP Adhesion Complex Regulates the PTEN/PI3K/AKT Signaling Pathway in Breast Cancer Cell Lines
Source: Int J Mol Sci. 2020 Jul 30;21(15):5458. doi: 10.3390/ijms21155458 (PMC7432699; doi:10.3390/ijms21155458)
Supplement: Supplementary file 1 [file ijms-21-05458-s001.zip › Supplemental figures/Supplemental Table 2 miRs targeting PINCH1.pdf]

Supplemental Table 2. ***miRs predicted to target PINCH1 identified using common 3 algorithms***

miRs	*miTG Score*	*Pct*	*Context+++ Score*	*mirSVR Score*
10a/b-5p		*<0.1*	*-0.17/-0.16*	*-0.5045*
17-5p		*<0.1*	*-0.02*	-0.4351
20a/b-5p		*<0.1*	*-0.02*	*-0.4351*
29a/b/c-3p	*0.84*	0.85	*-0.37*	*-0.9353*
30a/d	*0.90*	*<0.1*	*-0.27*	*-0.7883*
30b/c	*0.93*	*<0.1*	*-0.30*	*-0.7810*
30e	*0.87*	*<0.1*	*-0.05*	*-0.7956*
96-5p/1271-5p	*0.87*	*0.68*	*-0.11/-0.16*	*-0.1134*
101-3p	*0.90*	*<0.1*	*-0.23*	*-1.0419*
106a-5p		*<0.1*	*-0.02*	*-0.4351*
124-3p/506	*0.86*	*0.88*	*-0.34*	*-0.6398*
150-5p		*<0.1*	*-0.12*	*-0.5204*
182-5p	*0.99*	*0.15*	*-0.20*	*-0.1145/-0.8805*
186-5p		*N/A*	*-0.02*	*-0.2853*
190-5p		*<0.1*	*-0.13*	*-0.8379*
190b		*<0.1*	*-0.12*	-0.8450
192-5p	*0.91*	*<0.1*	*-0.43*	*-0.6686*
193a-3p	*0.85*	*<0.1*	*-0.36*	*-0.7517*
193b		*<0.1*	*-0.36*	*-0.7231*
202		*N/A*	*-0.16*	*-0.9608*
215-5p	*0.91*	*<0.1*	*-0.42*	*-0.6686*
340-5p	*0.95*	*N/A*	*-0.003*	*-0.1344*
376a/b-3p		*N/A*	*-0.17*	*-0.1467*
381-3p		*N/A*	*-0.08*	*-0.1967*
431-5p	*0.72*	*N/A*	*-0.14*	*-0.1868*
495-3p	*0.83*	*N/A*	*-0.02*	*-0.2485/-0.1264*

-*MicroT-CDS* (v5.0, updated July 2013): 0.7-1 or 0-1 scores predict higher possibility of targeting in the case of ***miTG Score***.
-*TargetScan* (released 7.2, March 2018): Probability of Conserved Targeting (***Pct***) as prediction scores and ***Context+++ Score*** is to be most negative.
-*miRanda* (released Aug. 2010): Scale values ≤0.1 predict higher possibility of PINCH1-targeting (***mirSVR***).
