# Supplementary figures and images for: MicroRNA-Dependent Targeting of RSU1 and the IPP Adhesion Complex Regulates the PTEN/PI3K/AKT Signaling Pathway in Breast Cancer Cell Lines
Source: Int J Mol Sci. 2020 Jul 30;21(15):5458. doi: 10.3390/ijms21155458 (PMC7432699; doi:10.3390/ijms21155458)

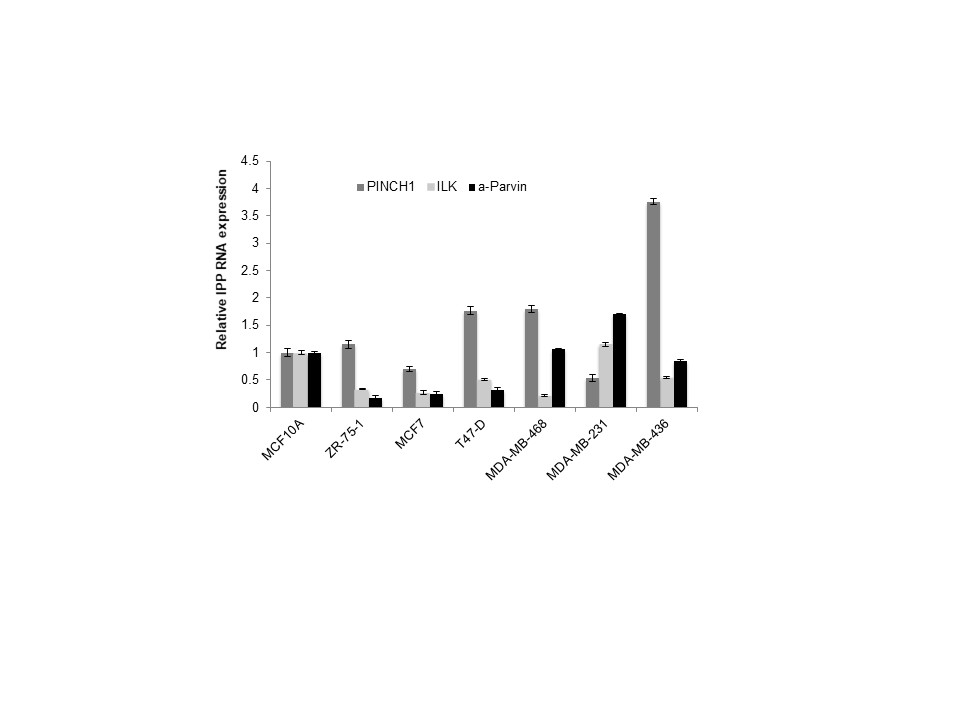

Supplement: Supplementary file 1 [file ijms-21-05458-s001.zip › Supplemental figures/Supplemental Figure 1.tif]
